# Emotional states in patients with cancer or with suspected oral potentially malignant disorders: a cross-sectional study in an oral medicine setting

**DOI:** 10.4317/medoral.26989

**Published:** 2025-03-23

**Authors:** Monica Bazzano, Rodolfo Mauceri, Giulia Marcon, Martina Coppini, Daniele Montemaggiore, Giuseppina Campisi

**Affiliations:** 1 Department of Precision Medicine in Medical, Surgical, and Critical Area (Me.Pre.C.C.), University of Palermo, Palermo, Italy; 2 Unit of Oral Medicine and Dentistry for fragile patients, Department of Rehabilitation, fragility, and continuity of care, University Hospital P. Giaccone, Palermo, Italy; 3 Department of Engineering, University of Palermo, Palermo, Italy; 4 Department of Biomedicine, Neurosciences and Advanced Diagnostics (BI.N.D.), University of Palermo, Palermo, Italy

## Abstract

**Background:**

This cross-sectional study aims to investigate the emotional states (i.e., anxiety, depression, and stress) of 4 distinct groups of patients attending an Oral Medicine setting.

**Material and Methods:**

four distinct subgroups of patients have been enrolled: 25 patients with OSCC (OSCC group); 25 patients with other solid tumor (ONCO group), 25 cancer patients with MRONJ (MRONJ group), 25 patients with oral potentially malignant disorder (OPMD group). Standardized assessment tools (i.e. Beck Depression Inventory (BDI) and Depression Anxiety Stress Scales-21 (DASS-21) questionnaires) were utilized to evaluate the patients’ emotional states.

**Results:**

Among the 100 patients enrolled, 59% of whom were female, and most patients were aged between 70 and 80 years. The mean total BDI score was 16.57, and the mean total DASS-21 score was 15.32. The mean scores of the OSCC group showed moderate depression, all the other groups were classified as mild depression. The OPMD group showed a significantly higher level of anxiety.

**Conclusions:**

Patients in the oral medicine setting may face various diseases and may develop emotional states that affect their treatment adherence. For effective management of these emotional states, the presence of a multidisciplinary team, including a psychologist, and the adoption of individualized approaches, seem to be necessary.

** Key words:**Dental anxiety, cancer, oral squamous cell carcinoma, psychological distress, BDI, Dass-21, osteonecrosis of the jaw, oral potentially malignant disorders.

## Introduction

Oral medicine and maxillofacial surgery are the main specialties dealing with severe and on occasions life-threatening orofacial disorders or their outcomes; therefore, an oral medicine setting may encourage states of fear and anxiety in patients (1). These setting often involve patients affected by oral potential malignant disorders (OPMD), or by oral squamous cell carcinoma (OSCC). Additionally, there are usually also patients affected by solid cancer with or without oral adverse drug reaction, such as medication-related osteonecrosis of the jaw (MRONJ). These patients often experience physical traumas and/or emotional disorders that can impact their quality of life (QoL), such as the fear of a recurrence. The latter may be a limiting factor hindering treatment adherence, care, and follow-up (2,3).

Emotional states contribute to shaping the ways in which we perceive, conceive, and express our reality. These inherently complex states delineate our thoughts, affective experiences, and undertaken behaviors. These epiphenomena are often linked to concomitant physiological, cognitive, and behavioral variations, which represent a synergistic outcome of the intricate interactions of biological, psychological (4). Moreover, social factors such as interpersonal relationships and the culture we are immersed in characterize the emotional experience and guide its manifestations (5).

Emotional states can be distinguished into "primary emotions," which are basic, innate, and universally shared emotions such as joy, sadness, anger, fear, and surprise, and "secondary emotions," emerging because of primary emotions and including feelings like love, hate, jealousy, and envy (6).

Personal perception and beliefs, moreover, can exert a significant influence on the development and manifestation of emotional states. For instance, the belief that an event is positive increases the likelihood of experiencing positive emotions. Also, emotional states influence behaviors, as seen when happiness prompts an approach-oriented attitude, and a smile directed towards others (7). Emotional states, ultimately, represent complex psychological realities, whose consequences manifest through physiological, cognitive, and behavioral changes, significantly influencing our interaction with the surrounding world and its inhabitants (8).

The diagnosis of cancer stems from a traumatic event that frequently elicits a diverse range of emotional reactions, including anxiety, fear, depression, anger, guilt, and isolation. Such emotional states can negatively impact the QoL for patients, their adherence to therapeutic protocols, and even the clinical course of the disease. Particularly, there are individuals with a predisposition to develop emotional disorders following a cancer diagnosis. Furthermore, specific characteristics of the disease, such as its severity, prognosis, and the type of anticipated treatment, can significantly influence the emotional state of patients (9-11).

It is known that anticancer therapies can generate a series of physical side effects, further contributing to the deterioration of patients' emotional well-being. Therefore, the social support provided by family, friends, and caregivers plays a crucial role in helping alleviate negative emotions related to the illness. Among the most common emotional disorders in cancer patients, anxiety, depression, and stress stand out. Anxiety presents as a natural response to stressful situations and may manifest through symptoms such as restlessness, agitation, and palpitations. Depression, on the other hand, constitutes a mood disorder with symptoms including sadness, loss of interest in daily activities, changes in sleep and appetite, and suicidal thoughts (12). Stress constitutes a physiological response to threatening or dangerous situations, whose symptoms may include an elevated heart rate, sleep deprivation, and irritability. Negative emotional states can interfere with therapeutic treatments in various ways. Firstly, they increase the risk of non-adherence to therapeutic protocols, resulting in an elevated risk of disease recurrence or progression. Secondly, they may contribute to the onset or worsening of side effects from anti-tumor therapies, such as cardiovascular diseases, gastrointestinal disorders, and sleep disturbances (13).

The objective of this cross-sectional study is to assess and to analyze the emotional states (i.e., anxiety, depression, and stress) of four distinct groups of patients attending an Oral Medicine setting.

Materials and Methods

- Study design

This observational study was approved by the local Institutional Ethics Committee of the University Hospital "Paolo Giaccone" of Palermo, Palermo, Italy (approval #1/2022).

The study was conducted according to the principles of the Declaration of Helsinki on experimentation involving human subjects, and written informed consent was obtained from all participants. The authors consecutively included 100 patients associated to different diseases/conditions, who attended the Unit of Oral Medicine at the “Paolo Giaccone” University Hospital in Palermo (Italy), from May to November 2022.

The patients recruited were 100 divided into 4 subgroups: 25 patients affected by OSCC (OSCC group); 25 patients with other solid tumor (ONCO group), 25 patients with other solid tumor and with MRONJ (MRONJ group), 25 patients with oral potentially malignant disorder (OPMD group).

- Eligibility criteria

1. age of patients ≥18 years;

2. patients affected by OSCC, or patients with other solid tumor, or patients with other solid tumor and with MRONJ or patients with OPMD;

3. the ability to read and understand the informed consent, and so to agree to participate to the study;

4. patients free by any known psychological disorder.

- Exclusion criteria

1. patients with cognitive deficits such that they cannot adequately complete the questionnaire and the visually impaired;

2. patients who are to undergo surgical treatment and/or biopsy, for reasons other than OPMD (e.g., salivary gland biopsy);

3. patients using benzodiazepines or other psychotropic medications.

- Description of tests used to assess emotional states.

All the patients recruited were administered the Beck Depression Inventory (BDI) and the Depression Anxiety Stress Scales-21 (DASS-21). The questionnaires were administered anonymously in a mixed paper-pencil and QR Code mode, and they were administered after the definitive diagnosis of the different diseases. The administering psychologist (M.B.) was present for questions and clarification.

The BDI is a self-administered instrument for assessing the severity of the affective and cognitive and motivational psychomotor and vegetative components of depression. It is a questionnaire that consists of 21 sets of statements describing various increasing levels of depressive symptomatology; each statement corresponds to a score, and these scores are administered to produce a total score.

Based on the total score there are different degrees of depression: minimal [0-13], mild [14-19], moderate [20-28], and severe [29-63] (14).

The present study also used the psychometric properties of the Italian version of the DASS-21 for its use in the Italian context. The DASS-21 allows the detection of three constructs: depression, anxiety, and stress. Depression includes dysphoria, hopelessness, devaluation of life, lack of interest/involvement, anhedonia, and inertia. Anxiety relates to autonomic nervous system arousal, skeletal muscle effects, situational anxiety, and subjective experience of anxious effects. Stress relates to the presence of chronic unspecific arousal levels, relaxation difficulties, nervous arousal, irritability, agitation, hyperactivity, and impatience (15).

Some questions regarding the medical history (e.g., smoking habit) were added to the original form of BDI and DASS-21.

A digital platform was created (by D.M.), which includes:

1. a section with two separate tests/forms "BDI, DASS-21" intended for patients, who filled them in totally anonymously way. To make everything smarter in filling in, a QR-code was created for each test in such a way that patients easily scanned with their cell phone camera and quickly filled in each questionary.

2. an "administrative" section, which included a Dashboard (giving an overview regarding the tests/forms filled out daily) and several subsections that allow statistical data to be extrapolated for each form based on the answers patients gave. The React framework for the front-end and Php and MySQL for the backend was used to implement the web platform.

- Outcome Measures

For each patient, the following data were recorded: demographic data, smoking habit, drug intake, the BDI scores according to the “An Inventory for Measuring Depression” (14), the DASS-21 scores according to the Italian version (16).

- Statistical Analysis

The analysis was carried out using the software R (R Core Team, 2022). Descriptive statistics were performed for the patients’ socio-demographic and physical characteristics. BDI and DASS-21 scores were summarized through mean value, standard deviation (SD), maximum, median and interquartile range (IQR). To analyze any statistically significant differences in anxiety between the patient groups, the data were initially tested to determine if any socio-demographic or physical characteristics were associated with the group membership by Chi-Square Test. The Shapiro-Wilk test was used to test the Normality distribution of BDI and DASS-21 scores.

In case of a lack of normality assumption, a non-parametric test was performed (e.g., Kruskal-Wallis’s test), to define if there was any significant difference between the average scores of questionnaires in the 4 groups.

The difference between BDI and DASS-21 scores in the groups was evaluated by the Kruskal-Wallis’s test to evaluate any statistically significant difference. The Dunn’s test was performed to identify which groups were different. The correlation analysis was also performed to study the strength of relation among questionnaires scores.

## Results

We enrolled 100 patients, there were more female than male (59% and 41%, respectively). Most patients were aged 70 to 80 years (29%). Female patients were the 68% of enrolled patients in the ONCO and OSCC groups. Conversely, in the MRONJ and OPMD groups, the percentage of female patients were slightly lower (59% and 64%, respectively). The age distribution varies among the groups. For instance, in the OSCC group, the most represented age ranges were 60-70 and 70-80 years (28% both), as well as the MRONJ group (44%). Most of the patients in the ONCO e OPMD group ranged between 50 and 60 years (40% and 32%, respectively).

Most of the patients enrolled have completed high school (32%) or achieved a degree (28%). Interestingly, most of the patients in the OSCC group have a low level of education (56%).

The OPMD group has the highest percentage of smokers (44%), while the ONCO group has the lowest percentage of former smokers (20%). No statistically significant differences were observed between groups concerning socio-demographic variables (Table 1), ensuring that any variations in BDI and DASS-21 scores were independent to these characteristics (Supplement 1).

**Figure 1:**
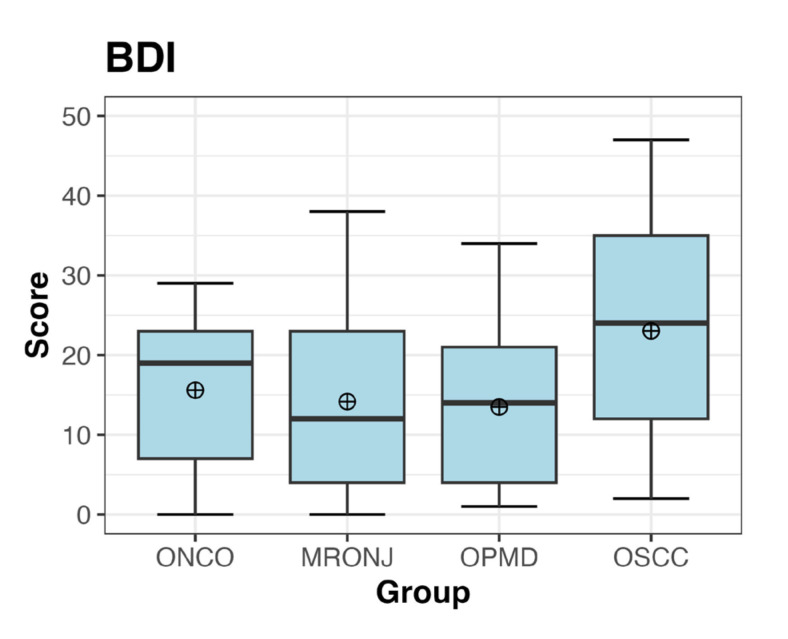
Boxplot describing the distribution and summary statistics of BDI scores for each group.

**Figure 2:**
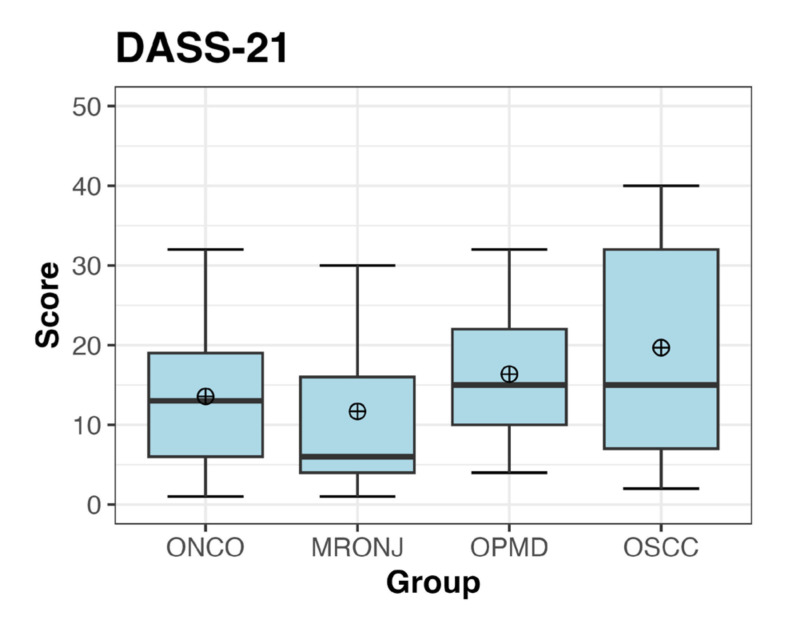
Boxplot describing the distribution and summary statistics of DASS-21 scores for each group.

Summary statistics of the BDI and DASS-21 scores are presented in Fig. 1 and Fig. 2, and detailed in Table 2, Table 3 and Table 4 (see also Supplement 1). Across all groups, the mean of the total BDI score was 16.57 ± 11.87, ranging from 13.48 (OPMD) to 23.04 (OSCC).

Based on the total score of BDI, the 44% of all the enrolled patients suffered of minimal depression. Furthermore, there were 17 patients affected by mild depression, 22 patients affected by moderate depression and 17 patients affected by severe depression. However, most of the patients suffering of severe depression were in the OSCC group (11/17 patients) (Supplement 2).

The Kruskal-Wallis test revealed significant differences in some BDI items, emphasizing the importance of considering these variations when tailoring patient care (Supplement 3). In detail, the most significant difference has been showed by the item “worthlessness”, the OSCC group reported the highest mean score 2.08 (*p* < 0.000). The OSCC group showed also the highest mean score in these items: “punishment” 1.32 (*p* < 0.009), “changes in sleeping” 1.72 (*p* < 0.037) and “changes in appetite” 1.16 (*p* < 0.015). However, regarding the BDI results on the items specifically related to sadness, pessimism, and loss of pleasure; each item revealed small distinctions among the four groups, even if the OSCC group showed the highest scores (Table 3).

The overall mean of the total DASS-21 items was 15.32 (±10.54). In general, the OSCC group exhibited also the highest mean DASS-21 score (19.7), indicating elevated levels of emotional challenges. Notably, while the OSCC group showed higher score in the item related to depression, the OPMD group showed higher scores in the item related to anxiety (particularly on items 9, 15, 19 and 20) and stress (particularly on items 6, 8 and 11) (Table 4).

The Shapiro-Wilk test highlighted that the BDI and DASS-21 overall scores were not normally distributed (Supplement 4). The application of the Kruskal-Wallis’s test highlighted significant differences between groups, however, it did not express which pairs of groups were different (*p-value*s equal to 0.0392 and 0.0599, respectively for BDI and DASS-21). Additionally, it underlined that such differences were statistically significant although weak, especially referring to DASS-21 (Supplement 3 and Suppement 5).

**Figure 3:**
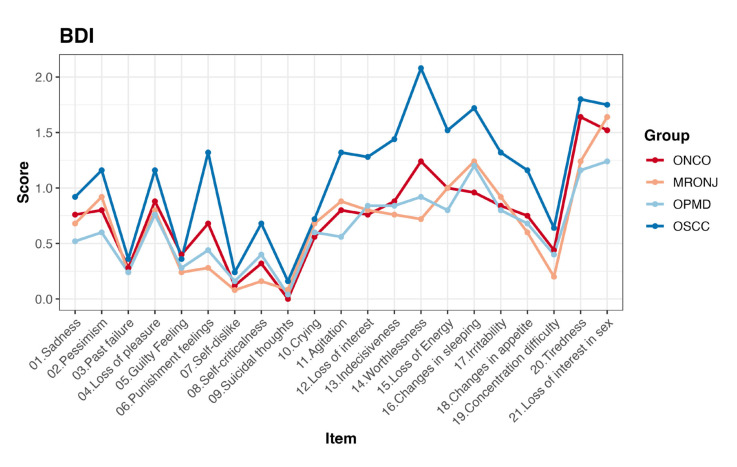
Average scores of BDI items of each group.

**Figure 4:**
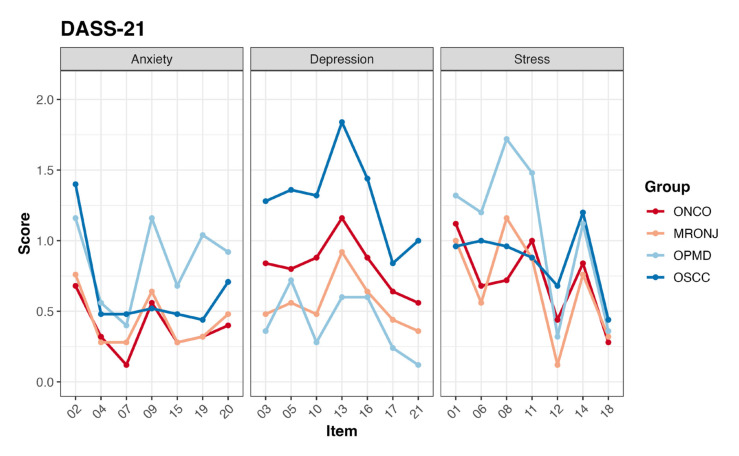
Average scores of DASS-21 items of each group.

The difference between groups was also tested for each item of both questionnaire BDI and DASS-21, results are displayed in Fig. 3 and Fig. 4.

Furthermore, we investigated the differences between the OPMD group and all the cancer patients of the OSCC, ONCO e MRONJ group. Patients with OPMD showed a lower level of depressive symptoms compared to the other three oncological groups (OSCC, ONCO and MRONJ), as evidenced by the consistency between the results obtained from the BDI and those from the DASS 21; however, they had a significantly higher level of anxiety (Supplement 6).

## Discussion

The present investigation represents a comprehensive and innovative attempt to explore the emotional states of patients in the oral medicine setting; indeed, the adopted approach aims to transcend the traditional boundaries of clinical research, typically centered on therapy and physical outcomes, investigating the rich complexity of emotional states associated to an oral medicine setting.

Moreover, the administration of the BDI and the DASS-21 contributed to outlining a comprehensive picture of the emotional challenges that patients may face during the clinical investigations.

The psychological well-being of patients has been evaluated by mean of two widely used scales (i.e., BDI and DASS-21). These scores provide valuable insights into the emotional and mental states of individuals within each group (Table 2, Table 3, and Table 4 and Fig. 3 and Fig. 4).

The BDI assesses various depressive symptoms, and understanding the distribution of these symptoms is crucial for tailoring effective interventions; so, the total BDI score provided an overview of overall depressive symptomatology in each group.

Notably, the OSCC group reported the highest mean BDI score [23], indicating a significant burden of depressive symptoms. This finding underscores the importance of psychosocial support for oral cancer patients. Indeed, patients with OSCC are subjected to intensive clinical treatments, such as demolitive surgery, radiotherapy of head and neck, and chemotherapy; all these therapies commonly cause significant side effects such as pain, fatigue and loss of appetite, strongly impairing their quality of life (17). Partial loss of the mouth can result in significant dysfunction of the stomatognathic system, impacting essential functions like speech, chewing, tasting, and swallowing. The latter may also profoundly affect the patients’ physical appearance, self-perception and socialization attitudes and behaviour (1).

Moreover, the reducing of the patients’ activities due to visible manifestations of the disease or unwanted effects of therapy may results in social isolation, that may contribute to an increase in the level of emotional distress. Such events may have a devastating emotional impact, leading to problems with self-esteem and depression (9).

There are several studies on the depression of patients with different cancer (e.g., breast, lung, prostate) evaluated with the BDI questionnaire, the results show value of minimal depression, close to the level of mild depression (18-20). The latter is comparable to the data of the ONCO and MRONJ group of the present study.

Dzebo *et al*. investigated the depression of patients with oral cavity cancer; however, their results regarding the BDI differs from the present study. In detail, up to 56% of their patients do not suffer of depression (“minimal” depression) according to the BDI scores, while only the 35 % of patients suffered of moderate or severe depression (16% and 19%, respectively)(21). In the OSCC group of the present study the result was slightly different, indeed only the 32% of patients were suffering of minimal depression, while the 52% of patients with OSCC showed moderate or severe depression (8% and 44%, respectively).

Conversely, the OPMD group had a slightly lower mean BDI score (13.5), suggesting relatively lower depressive states, even in the absence of a definitive diagnosis of OPMD or OSCC. Indeed, while the mean scores of the BDI classifies the OSCC group at a level of moderate depression, all the other groups are classified as mild depression. These observations are consistent with a previous multicenter study, in which patients affected by OMPD (i.e., oral lichen planus) showed normal or mild depressive scores (50.7% and 39.3%, respectively). However, it must be considered that in this study the depression was evaluated by a different questionnaire (i.e., Hamilton Rating Scale for Depression)(22).

The latter highlights the importance for healthcare professionals to consider not only the physical aspects of therapy but also the emotional and psychological impacts. Creating supportive listening environments can help patients with OSCC to manage both their disease and medication side effects (23).

The DASS-21 composite score provides an overview of emotional distress, evaluating the patients’ depression, anxiety, and stress. This aspect should deserve more attention during the diagnostic assessment. In our clinical practice, patients affected by OPMD often exhibit a constant state of agitation during their visits in an oral medicine setting. This anxiety is closely linked to the need for thorough diagnostic investigations to exclude the presence of OSCC. The awareness of potential malignant lesions induces emotional hyperarousal in patients, who fear receiving negative news about their health (24). Therefore, in the management of patients with suspected OPMD, it is crucial to carefully consider the emotional and psychological aspects of the patients.

Additionally, in the above mentioned Italian multicenter study, most of the patients affected by OPMD (i.e., oral lichen planus) showed normal or mild anxiety scores (47.3% and 40.7%, respectively). As previously stated, even for these data it must be considered that a different questionnaire was administered (i.e., Hamilton Rating Scale for Anxiety) (22).

The BDI and DASS-21 scores complement each other. While the BDI focuses specifically on depressive symptoms, the DASS-21 captures a broader spectrum of emotional experiences.

The analysis of the results obtained from the BDI and DASS-21 tests among the four patient groups reveals interesting differences in levels of depression, anxiety, and stress.

The OPMD group stands out for presenting significantly higher scores in various elements of both tests. highlighting higher level of anxiety and stress. Once again, these results suggest a noTable emotional and psychological vulnerability among patients with OPMD.

The OSCC group shows higher scores for depression compared to the other groups. The items "worthlessness" and "changes in sleeping" in the BDI, along with the specific item on depression in the DASS-21, are commonly associated with patients' moods, particularly depression.

A sense of worthlessness is a key symptom of depression, in which the person may feel a deep sense of worthlessness or purposelessness. This can manifest through negative thoughts about oneself, feelings of guilt and self-criticism and can greatly affect daily functioning. Sleep changes are also a common feature of depression. People with depression may experience sleep disorders such as insomnia (difficulty falling asleep or maintaining sleep) or hypersomnia (excessive daytime sleepiness). These changes in sleep can have a significant impact on overall well-being and ability to function during the day (21).

As previously mentioned, OSCC diagnosis and subsequent treatments can have a significant impact on patients' emotional well-being, as demonstrated by the results of the BDI and DASS-21 tests conducted on our patients with OSCC. Fully understanding the emotional challenges patients face allows healthcare providers to optimize care, promote mental health, and improve treatment overall (10,11).

Everyone responds uniquely to trauma and develops different emotional challenges; however, our research has highlighted that different groups of patients in oral medicine experience similar emotional difficulties. Assessing and supporting a trauma patient is not an easy task and may require the intervention of a psychologist (25).

Standard care by a psychologist and/or the adoption of innovative approaches could prove useful for patients in the context of oral medicine, opening new perspectives in the global management of cancer patients (26). For example, the graphic novel seems to be an innovative strategy to reduce the anxiety of patients waiting to undergo a biopsy to diagnose an OPMD and to support them to wait for a potential bad news (2).

Oral health isn't just about the health of teeth and periodontium; it is often related to other oral problems. Therefore, it is important to assume a holistic approach in oral medicine.

Currently, a multidisciplinary approach is recommended to treat various categories of patients, personalize interventions and maximize clinical outcomes, as well as improve emotional well-being during the treatment journey. This approach is particularly advantageous for patients with OSCC (27-29).

A multidisciplinary team of experts, including not only dentists but also professionals who can communicate effectively, can develop targeted educational programs that meet the specific needs of patients. The main goal should be to improve overall oral health but also to ensure a better quality of life for patients. Indeed, these patients may experience several emotional states impacting their QoL and leading to lower adherence to treatments and regular check-up. Indeed, it could be recommended for these groups of patients a cognitive behavioral psychotherapy support to learn how to manage anxiety, depression and/or distress with different techniques such as body relaxation, cognitive restructuring, and systematic desensitization (27-29).

Considering the results, to date raising the awareness about this issue and promoting health education is essential. The scientific community should further explore the complex interconnections between emotional states, dental treatments, and oncological conditions, paving the way for future research and innovative approaches in oral medicine.

Emotional states are influenced by multiple factors, including individual, family, social, and psychological aspects that can be difficult to isolate and evaluate in one study.

There are other potential limitations to be considered. Since the patients are all Caucasian and born in the South of Italy, this limits the generalizability of the results and makes them less representative of the entire population, since cultural and socioeconomic differences between different countries and ethnic groups are not considered.

Additionally, considering that we recruited patients with OPMD only from the South of Italy, as showed by another Italian multicenter study on the depression and anxiety of patients affected by OLP, it must be considered that patients of the Central-South of Italy presented statistically higher levels of anxiety, depression and sleep disturbances compared to the Northern group (30).

## Conclusions

Emotional states can have a significant impact on patients, with consequences on treatment adherence and outcomes. Adopting individualized approaches for anxiety management, based on an understanding of specific traumas that generate anxiety in patients, can contribute to enhancing the overall quality of oral care. Hopefully, further research may promote the knowledge on emotional states of patients in oral medicine settings and develop effective interventions to reduce their anxiety.

## Figures and Tables

**Table 1:** Descriptive statistics relating to patient socio-demographic and physical characteristics.

Characteristics		N	OSCC (n.25 pts)		ONCO(n.25 pts)		MRONJ(n.25 pts)		OPMD (n.25 pts)		Chi - SquareTest
			N	%	N	%	N	%	N	%	Statistic(p-value)
Gender	Female	59	9	36	16	64	17	68	17	68	7,40 (0.06)
	Male	41	16	64	9	36	8	32	8	32	
Age	(25;40)	3	0	0	1	4	1	4	1	4	20.02 (0.17)
	(40;50)	13	3	12	3	12	1	4	6	24	
	(50;60])	26	3	12	10	40	5	20	8	32	
	(60;70)	20	7	28	5	20	4	16	4	16	
	(70;80)	29	7	28	6	24	11	44	5	20	
	80 +	9	5	20	0	0	3	12	1	4	
Educational level	Primary school	15	7	28	0	0	5	20	3	12	10.04 (0.35)
	Middle School	24	7	28	6	24	6	24	5	20	
	High school	32	6	24	10	40	7	28	9	36	
	Degree	28	5	20	9	36	6	24	8	32	
	NA	1	0	0	0	0	1	4	0	0	
Smoking habit	smoker	31	9	36	5	20	6	24	11	44	15.064 (0.02)
	ex-smoker	41	14	56	10	40	7	28	10	40	
	never smoked	28	2	8	10	40	12	48	4	16	

**Table 2:** Summary statistics of the BDI and DASS-21 scores.

	BDI					DASS-21				
	Mean	SD	Min-Max	Median	IQR	Mean	SD	Min-Max	Median	IQR
OSCC	23	13.9	2-47	24	23	19.7	13.7	2-40	15	25
ONCO	15.6	9.73	0-29	19	16	13.6	8.93	1-32	13	13
MRONJ	14.2	11.5	0-38	12	19	11.7	9.97	1-30	6	12
OPMD	13.5	10.3	1-34	14	17	16.4	7.55	4-32	15	12

**Table 3:** Mean and standard deviation for each BDI item and total score.

Item	Total		OSCC		ONCO		MRONJ		OPMD		Kruskal-Wallis Test		
	Mean	SD	Mean	SD	Mean	SD	Mean	SD	Mean	SD	W	*p-value*	
Sadness	0.72	0.60	0.92	0.81	0,76	0.52	0.68	0.48	0.52	0.51	4.3	0.234	-
Pessimism	0.87	0.82	1.16	1.03	0.80	0.65	0.92	0.91	0.60	0.58	4.2	0.237	-
Past failure	0.28	0.62	0.36	0.76	0.28	0.68	0.24	0.52	0.24	0.52	0.2	0.970	-
Loss of pleasure	0.90	0.76	1.16	0.99	0.88	0.60	0.80	0.76	0.76	0.60	2.8	0.420	-
Guilty Feeling	0.32	0.57	0.36	0.64	0.40	0.58	0.24	0.52	0.28	0.54	1.6	0.660	-
Punishment feelings	0.68	1.01	1.32	1.28	0.68	1.03	0.28	0.54	0.44	0.77	11.6	0.009	**
Self-dislike	0.15	0.44	0.24	0.66	0.12	0.33	0.08	0.28	0.16	0.37	1.0	0.798	-
Self-criticism	0.39	0.76	0.68	1.18	0.32	0.63	0.16	0.37	0.40	0.58	2.9	0.401	-
Suicidal thoughts	0.07	0.29	0.16	0.37	0.00	0.00	0.08	0.41	0.04	0.20	6.1	0.109	-
Crying	0.64	0.73	0.72	0.89	0.56	0.65	0.68	0.80	0.60	0.58	0.2	0.972	-
Agitation	0.89	0.89	1.32	1.11	0.80	0.65	0.88	0.97	0.56	0.58	7.0	0.072	.
Loss of interest	0.92	0.99	1.28	1.24	0.76	0.78	0.80	1.00	0.84	0.85	2.7	0.448	-
Indecisiveness	0.98	1.00	1.44	1.26	0.88	0.78	0.76	1.05	0.84	0.75	5.3	0.154	-
Worthlessness	1.24	1.16	2.08	1.08	1.24	1.13	0.72	1.06	0.92	0.95	19.8	0.000	***
Loss of Energy	1.08	1.13	1.52	1.29	1.00	0.96	1.00	1.26	0.80	0.87	4.4	0.224	-
Changes in sleeping	1.28	0.90	1.72	0.98	0.96	0.54	1.24	0.97	1.20	0.91	8.5	0.037	*
Irritability	0.97	0.92	1.32	1.14	0.84	0.69	0.92	0.91	0.80	0.82	3.3	0.353	-
Changes in appetite	0.80	0.82	1.16	0.75	0.75	0.61	0.60	0.87	0.68	0.95	10.5	0.015	*
Loss of concentration	0.42	0.57	0.64	0.76	0.44	0.51	0.20	0.41	0.40	0.50	5.9	0.118	-
Tiredness	1.46	1.07	1.80	1.00	1.64	1.08	1.24	1.13	1.16	0.99	6.7	0.082	.
Loss of interest in sex	1.54	1.28	1.75	1.36	1.52	1.23	1.64	1.25	1.24	1.30	2.3	0.518	-
BDI Total	16.57	11.87	23.04	13.63	15.60	9.55	14.16	11.27	13.48	10.09	8.36	0.039	*

Signif. codes: 0 ‘***' 0.001 ‘**' 0.01 ‘*' 0.05 ‘.' 0.1 ‘-' 1.

**Table 4:** Mean and standard deviation for each DASS-21 item and total score.

Item		Total		OSCC		ONCO		MRONJ		OPMD		Kruskal-Wallis Test		
		Mean	SD	Mean	SD	Mean	SD	Mean	SD	Mean	SD	W	*p-value*	Test
Anxiety	2	1.00	0.90	1.40	1.04	0.68	0.80	0.76	0.83	1.16	0.75	10.0	0.019	*
	4	0.41	0.60	0.48	0.71	0.32	0.63	0.28	0.46	0.56	0.58	4.4	0.226	-
	7	0.32	0.55	0.48	0.77	0.12	0.33	0.28	0.46	0.40	0.50	5.2	0.155	-
	9	0.72	0.79	0.52	0.82	0.56	0.65	0.64	0.76	1.16	0.80	10.6	0.014	*
	15	0.43	0.57	0.48	0.65	0.28	0.46	0.28	0.46	0.68	0.63	7.9	0.049	*
	19	0.53	0.59	0.44	0.65	0.32	0.48	0.32	0.48	1.04	0.45	26.3	0.000	***
	20	0.63	0.79	0.71	1.00	0.40	0.58	0.48	0.71	0.92	0.76	7.2	0.066	*
Depression	3	0.74	0.92	1.28	1.24	0.84	0.75	0.48	0.71	0.36	0.57	11.6	0.009	**
	5	0.86	0.97	1.36	1.22	0.80	0.82	0.56	0.82	0.72	0.84	7.2	0.065	.
	10	0.74	0.89	1.32	1.11	0.88	0.78	0.48	0.71	0.28	0.54	17.8	0.000	***
	13	1.13	1.00	1.84	1.14	1.16	0.99	0.92	0.64	0.60	0.76	17.7	0.001	***
	16	0.89	1.00	1.44	1.29	0.88	0.88	0.64	0.81	0.60	0.76	7.7	0.052	.
	17	0.54	0.81	0.84	1.07	0.64	0.81	0.44	0.71	0.24	0.44	5.2	0.158	-
	21	0.51	0.73	1.00	1.00	0.56	0.65	0.36	0.49	0.12	0.33	15.5	0.001	***
Stress	1	1.10	0.77	0.96	0.89	1.12	0.67	1.00	0.82	1.32	0.69	4.0	0.267	-
	6	0.86	0.84	1.00	0.96	0.68	0.80	0.56	0.71	1.20	0.76	9.4	0.024	*
	8	1.14	0.74	0.96	0.54	0.72	0.74	1.16	0.75	1.72	0.54	24.9	0.000	***
	11	1.06	0.73	0.88	0.67	1.00	0.71	0.88	0.80	1.48	0.59	11.6	0.009	**
	12	0.39	0.69	0.68	1.03	0.44	0.65	0.12	0.33	0.32	0.48	5.7	0.125	-
	14	0.98	0.90	1.20	1.04	0.84	0.85	0.76	0.88	1.12	0.78	4.1	0.247	-
	18	0.35	0.72	0.44	0.87	0.28	0.68	0.32	0.69	0.36	0.64	0.5	0.925	-
DASS-21 Total		15.32	10.54	19.68	13.48	13.56	8.75	11.68	9.78	16.36	7.40	7.41	0.060	.

Signif. codes: 0 ‘***' 0.001 ‘**' 0.01 ‘*' 0.05 ‘.' 0.1 ‘-' 1.
